# Knowledge of midwives about hypertensive disorders during pregnancy in primary healthcare

**DOI:** 10.4102/phcfm.v8i1.899

**Published:** 2016-04-11

**Authors:** Ethelwynn L. Stellenberg, Nompumelelo L. Ngwekazi

**Affiliations:** 1 Division of Nursing, Stellenbosch University, South Africa

## Abstract

**Background:**

Many factors or medical conditions may influence the outcome of pregnancy, which in turn, may increase infant and maternal morbidity and mortality. One such condition is an increase in blood pressure (BP).

**Setting:**

The study was conducted in maternity obstetrical units (MOUs) in primary healthcare clinics (PHCs) in the Eastern Cape, South Africa.

**Objectives:**

To determine the knowledge about hypertensive disorders during pregnancy (HDPs) of registered midwives working in MOUs in PHCs.

**Methods:**

A quantitative descriptive correlation research design was applied. A simple random sample of 43 (44%) rural and urban clinics was selected, and all registered midwives (*n* = 101) working in these clinics completed a self-administered questionnaire.

Data were collected over a period of 1 month. The reliability and validity of the methodology were supported by experts and a pilot study. Descriptive statistics including various statistical tests to determine any associations between variables using a 95% confidence interval were applied.

**Results:**

A gap in the knowledge of midwives about HDPs was identified. Only 56.4% of the participants correctly answered the questions on the clinical manifestations of severe pre-eclampsia and 68.3% on the factors affecting BP, whereas 27.7% had no understanding about pre-eclampsia. Significant statistical differences were identified in the knowledge of staff in clinics where doctors visit regularly versus those in clinics where there are no visits (*p* = 0.04), and between experience of midwives and management of HDPs (*p* = 0.02).

**Conclusion:**

The knowledge of midwives is deficient regarding HDPs. Continuous professional development is critical in midwifery both in theory and in clinical practice.

## Introduction

### Social value

It is imperative that midwives working in primary healthcare have the required knowledge about specific avoidable conditions during pregnancy. This will enable them to assess, diagnose and manage the pregnant patient efficiently and effectively and ensure that infant and maternal morbidity and mortality rates are kept at a minimum. Globally, much has been achieved over a period of 20 years, with global maternal deaths decreasing from 400 to 210 per 100 000 live births from 1990 to 2010. However, the target of reducing maternal mortality by 75% will still require much effort and expedited political support. Providing good antenatal care will ensure a reduction in the child mortality rate – Millennium Development Goal 4.^1^

The maternal death rate in South Africa for the period 2011–2012 due to avoidable deaths of pregnant women was similar to the 2008–2010 rate, accounting for 66.3% of avoidable deaths, of which hypertension contributed 16.5%. In the South African region where the research was conducted, the percentage of avoidable deaths due to hypertensive disorders during pregnancy (HDPs) was 18.8.^2^

A case study completed for the confidential enquiry into maternal deaths in South Africa reports that healthcare provider problems contributed to 14% – 38% of maternal deaths, with most of them occurring in district and regional hospitals. The factors that contributed included not assessing patients properly, delays in referral, failure to recognise the problem, not following standard protocols and poor monitoring.^3^

Further results of the study showed that the 507 deaths associated with HDPs in South Africa during the period 1999–2001 were due to avoidable medical factors such as mismanagement of HDPs, delay in referral, unavailability of transport, lack of protocol for the management of eclampsia and failure to follow clinical protocols of care. In addition, the same author identified in a population-based study in Durban that hypertensive disorders, which contributed to 12% of all cases, were aggravated by various issues such as administrative problems, lack of transport to clinics and barriers to access to healthcare facilities, as well as failure to terminate pregnancy and lack of appropriately trained staff.^4^ In a study conducted to improve neonatal survival in South Africa, the researchers found that the rate of death during the first week of life for infants weighing 1000 g or more was ‘unacceptably’ high, 8.7/1000, with rural death rates as high as 10.42/1000. The factors contributing to this high infant mortality rate were intra-partum hypoxia and preterm delivery, aggravated further by modifiable factors such as inadequate staffing and facilities, poor care during labour and poor neonatal resuscitation and basic care.^5^

Consequently, specific guidelines were introduced in South Africa for all low-risk antenatal care to improve the quality of care during and after pregnancy and reduce maternal deaths. This service is provided by midwives in primary healthcare clinics (PHCs) and at 24-hour comprehensive obstetric units, referred to as midwifery obstetrical units (MOUs).^6^ ‘A midwife is a person who is qualified and competent to independently practise midwifery in the manner and to the level prescribed and who is capable of assuming responsibility and accountability for such practice’.^7^ Currently, midwifery is offered (1) as a basic 1-year diploma programme, (2) as part of the 4-year degree or diploma programmes, and (3) at the post-graduate level as an advanced course.^8,9,10^

Midwives are thus responsible for ensuring that the obstetric service provided is effective and efficient enough to bring about a reduction in infant and maternal mortality rates. To provide such a service, the knowledge and competence of midwives are critical. The purpose of this article is therefore to describe the knowledge of midwives delivering a service in MOUs in primary healthcare specifically about HDPs.

### Scientific value

HDPs are amongst the major causes of maternal and perinatal morbidity and mortality.^11^ Pre-eclampsia is the most common medical condition that occurs during pregnancy, complicating 10% of pregnancies. Worldwide between 50 000 and 70 000 maternal deaths occur annually because of severe pre-eclampsia.^12^

HDPs are classified into four categories, namely (1) chronic hypertension, (2) pre-eclampsia/eclampsia, (3) pre-eclampsia superimposed on chronic hypertension and (4) gestational hypertension.^13^ However, midwives and other maternity care providers use the term ‘hypertensive disorders of pregnancy’ to describe a range of hypertensive conditions.^14^

Strategies designed in the Netherlands emphasise that the midwife is the primary contact who should manage all uncomplicated antenatal conditions without the supervision of the obstetrician but must refer to the next level should complications be identified.^15^

In England, pregnant women are managed at midwifery units and are only transferred to obstetric units in hospitals if complications arise.^16^ The findings of a telephonic survey revealed that a midwife as the primary contact is valued by women attending the clinic. Despite the fact that positive feedback was received from women who attended this particular antenatal clinic, one woman indicated that the service was poor and it appeared that the midwife was uncertain and seemed not to know what she was doing.^17^

The role of the midwife in the PHC is critical in preventing, identifying and managing hypertension disorders. In the event that pre-eclampsia is diagnosed, the patient is immediately referred to an obstetrician.^18^ Consequently, comments that poor or substandard care was delivered will be avoided.

Blood pressure (BP) control during pregnancy is crucial for the safety of both mothers and babies.^14,18^ BP values during pregnancy have been shown to be associated with a continuous inverse effect on foetal growth.^19^

It was identified that student midwives were unable to manage maternal deterioration in clinical practice, which may gravely influence the maternal morbidity and mortality rate. This was confirmed in a study that assessed student midwives in a simulated setting, which showed that as the patient deteriorated, with marked changes in vital signs such as BP, the ‘key observations’ of the student midwives decreased.^20^

Hypertension in pregnancy causes reduced blood flow to the placenta, which results in anoxia and a limited supply of nutrients to the growing foetus, leading to slow growth and premature separation of the placenta from the uterine walls, causing severe bleeding.^21^ Further studies^11,22,23^ validate these results as the outcome of HDPs has a major influence on neonatal outcomes. Adverse pregnancy outcomes include still birth, intrauterine death, intrauterine growth restriction and termination of pregnancy.

Thus, early diagnosis is of utmost importance to identify any pathology. An early diagnosis could improve the pregnancy outcome because better maternal and foetal monitoring would lead to earlier detection of the clinical signs of the disease and, where necessary, medication can be given.^24^ In South Africa, the majority of pregnancy-related care is delivered by midwives at MOUs based at the primary healthcare level.^6^

Because of this critical role the midwife has in primary healthcare, the aim of this study was to assess the midwives’ knowledge about HDPs, including assessment, diagnosis, and management.

It was against this background that it became essential to conduct a scientific study to determine the knowledge of midwives about HDPs. A deficit in the knowledge of registered midwives about HDPs, including its assessment, diagnosis and management, could result in problems such as misdiagnosis and delayed referrals. This could contribute to high maternal and infant morbidity and mortality rates.

### Aim and objectives

To determine the knowledge about HDPs of registered midwives working in MOUs in PHCs.

## Research methods and design

### Study design

A quantitative descriptive correlational non-experimental research design was applied in this study.

### Setting

The study was undertaken in rural and urban PHCs in the Eastern Cape.

### Study population sampling strategy

Pregnancy-related services are provided in 98 rural and urban PHCs. Using a simple random sampling method, *n* = 43 (44%) clinics, of which 28 were rural and 15 urban, were chosen. All 101 registered midwives who practise in these clinics participated in the research.

### Specific criteria

Registered midwives working in the clinics identified for the purpose of this study in the Eastern Cape were eligible to participate in the study.

### Pilot study

A pilot study that included a random sample of 10 (10%) based on the actual size of the population (*>N*> = 101) of the main study was conducted in four of the PHCs (*>N*> = 101) of the main study. The results of the pilot study did not form part of the main study.

### Data collection

A 34-item multi-choice questionnaire was developed based on the objective of the study and the scientific literature on HDPs. The instrument consisted of four sections, namely demographic information and multiple-choice questions on hypertensive disorders and their assessment, diagnosis and management. After written informed consent was obtained, the researcher personally distributed the questionnaire in an envelope to the midwives and collected the completed questionnaire in the sealed envelope; completion of the questionnaire did not exceed 30 min.

### Reliability and validity

A statistician was consulted about the design of the questionnaire and planning of the data analysis, who rendered support throughout the study to ensure the correctness and accuracy of the study. Midwifery experts and a research methodologist were consulted to evaluate the content validity of the questionnaire. The pilot study further supported the reliability and validity of the study.

### Data analysis

Guided by a statistician, descriptive analysis, including statistical tests such as chi-squared, Mann-Whitney, Kruskal-Wallis and Spearman correlation, was applied to test for any statistical associations between the demographic variables and the responses to the questions about the knowledge of midwives regarding HDPs.

### Ethical considerations

Consent was obtained from the Eastern Cape Department of Health, clinics and Human Science Committee at the Faculty of Medicine and Health Sciences, Stellenbosch University (N08/06/178).

Informed written consent was obtained from all the participants. Confidentiality and anonymity were assured. The participants were informed about their right to withdraw from the study without any repercussions.

All data were managed confidentially and were only accessible to the researcher and the supervisor; the data will be destroyed after a period of 5 years.

## Results

### Biographical data

The majority of the 101 participants were older than 40 years of age (67, 66.3%) and female (88, 87.1%). Most of them (74, 73.3%) were experienced midwives with more than 3 years of experience, and 41 (40.6%) had more than 10 years’ experience. Only 5 (5.0%) were newly qualified (6 months or less). Most participants (53, 52.4%) indicated that they had a 1-year diploma in midwifery, followed by those with a 4-year diploma in general nursing (Psychiatry & Community Health) and Midwifery (33, 33.7%). Only one participant held an advanced diploma qualification in midwifery. Most of the participants (73, 72.3%) indicated that a doctor regularly visited the clinic, whereas 28 (27.7%) indicated that they do not receive any visits from doctors.

### Knowledge about hypertensive disorders during pregnancy

Table 1 shows the number of participants who correctly answered questions related to specific aspects of HDPs. The results showed that many participants were not able to define hypertension (33.7%), gestational hypertension (43.6%) or chronic hypertension during pregnancy (29.7%). Furthermore, 27.7% had no understanding about pre-eclampsia, 43.6% did not associate obesity as a risk factor with the development of pre-eclampsia and 36.6% had no knowledge about the effects of pre-eclampsia on the mother.

**TABLE 1:** Knowledge about hypertensive disorders (*N* = 101).

Questions related to aspects of HDPs	Correct *n* (%)
Definition of hypertension in pregnancy	67 (66.3)
Definition of gestational hypertension	57 (56.4)
Definition of chronic hypertension	71 (70.3)
Definition of eclampsia	98 (97.0)
Understanding of pre-eclampsia	73 (72.3)
Major risk factor associated with development of pre-eclampsia	57 (56.4)
Maternal effects of pre-eclampsia	64 (63.4)
Foetal effects of eclampsia	90 (89.1)
Definition of proteinuria	61 (60.4)

*Source*: Stellenberg and Ngwekazi (2016) HDPs, hypertensive disorders during pregnancy.

### Assessment and diagnosis of hypertensive disorders during pregnancy

Knowledge scores for the diagnosis and assessment of hypertensive disorders are shown in Table 2. Only 56.4% of the participants responded correctly to the question about the clinical manifestations of severe pre-eclampsia and the factors influencing BP, and 61.4% responded incorrectly to the appropriate position of the patient for correct reading of BP.

**TABLE 2:** Assessment and diagnosis of HDPs (*N* = 101).

Questions related to aspects of HDPs	Correct *n* (%)
Subjective data pertaining to medical history	79 (78.2)
Social history of pregnant women	73 (72.3)
Clinical manifestations of pre-eclampsia	96 (95.0)
Clinical manifestations of severe pre-eclampsia	57 (56.4)
Assessment of oedema in pregnant women with hypertension	92 (91.1)
Understanding pitting oedema	89 (88.1)
Factors affecting blood pressure reading	69 (68.3)
Appropriate patient position for correct reading of blood pressure	39 (38.6)
Using the same position or not when measuring the blood pressure of a pregnant woman	87 (86.1)
Type of test for pre-eclampsia	95 (94.0)
Test for assessing foetal status	76 (75.2)
Laboratory tests to diagnose pre-eclampsia	100 (99.0)

*Source*: Stellenberg and Ngwekazi (2016) HDPs, hypertensive disorders during pregnancy.

### Management of hypertensive disorders in pregnancy

Knowledge scores related to the management of HDPs are shown in Table 3. Only 28.9% responded correctly with reference to the prevention of pre-eclampsia, only 33.7% were able to advise the type of relaxation suitable for pregnant women with hypertension and only 26.7% responded correctly about the route to be used for the administration of magnesium sulphate.

**TABLE 3:** Management of hypertensive disorders in pregnancy (*N* = 101).

Questions related to aspects of the management of HDPs	Correct *n* (%)
Prevention of pre-eclampsia	29 (28.9)
Advice to women with pre-eclampsia regarding their diet	83 (82.2)
Type of suitable relaxation for pregnant women with hypertension	34 (33.7)
Encouraging bed rest	85 (84.2)
Consultation by midwife	60 (59.4)
Treatment of pre-eclampsia	96 (95.0)
Route to be used for the administration of magnesium sulphate	27 (26.7)

*Source*: Stellenberg and Ngwekazi (2016) HDPs, hypertensive disorders during pregnancy.

### Factors associated with knowledge scores

Table 4 shows the relationship between the knowledge of midwives and key underlying variables such as gender, qualifications and support from a doctor. Male midwives accoucheur just had significantly better knowledge of the management of HDPs (*p* = 0.05), but otherwise gender was not significant. Midwives who had no regular support from a doctor had a significantly better total knowledge score. There was no correlation between age and any of the knowledge scores, but greater experience was correlated, on the one hand, with better knowledge of HDPs (Spearman coefficient 0.28, *p* < 0.01) and, on the other, with worse knowledge of its management (Spearman coefficient −0.24, *p* = 0.02).

**TABLE 4:** Results showing the association between knowledge scores and key variables.

Factor	*N*	Mean scores			

		Total knowledge	Knowledge about HDPs	Assessment and diagnosis	Management HDPs
**Gender**					
Male	13	0.683	0.504	0.807	0.605#
Female	88	0.663	0.551	0.787	0.512#
**Qualifications**					
4-Year diploma	33	0.664	0.538	0.784	0.534
4-Year degree	14	0.659	0.507	0.785	0.544
1-Year basic diploma in midwifery	53	0.669	0.555	0.794	0.514
**Regular doctor visits to clinics**					
No	28	0.691	0.587	0.808	0.544
Yes	73	0.656	0.529	0.782	0.517

*Source*: Stellenberg and Ngwekazi (2016) HDPs, hypertensive disorders during pregnancy.

## Discussion

### Key findings

This study identified wide gaps in the knowledge of midwives about HDPs, including their assessment, diagnosis and management. This is a major concern as hypertension contributed to 16.5% of maternal deaths in South Africa, and in the particular region where the research was conducted, 18.8% of maternal deaths were from hypertension.^2^ Globally, severe pre-eclampsia and eclampsia contribute to 50 000–70 000 maternal deaths annually.^12^

The overall majority of participants had more than 3 years of experience, which included participants with more than 10 years of experience in clinical midwifery practice. According to the description of the third stage in a five-stage theory on competence, from novice to expert, an individual is considered competent with 2–3 years of experience. Most of these participants should thus have been competent according to this theory, and participants with more than 10 years of experience are regarded as experts in the field.^25^ The participants were thus not junior practitioners and should have been an asset to the PHCs and MOUs in clinical practice and knowledge about midwifery practice, specifically in the area where the research was conducted.

In clinics where doctors visited for consultation purposes, the response results of the participants were much lower in all sections, namely knowledge, assessment and diagnosis as well as the management of HDPs, compared with clinics where there were no doctors. A midwife is defined as a person who is ‘qualified and competent to independently practise midwifery in the manner and to the level prescribed and who is capable of assuming responsibility and accountability for such practice’.^7^

### Discussion of key findings

It is problematic if midwives functioning independently at the PHC level are not able to distinguish between the various categories of HDPs, diagnose, assess or manage patients with hypertension. In this study, despite the fact that all the participants were fully qualified midwives, the majority had no knowledge about the appropriate position for correct reading of BP.

It cannot be emphasised enough that BP control during pregnancy is crucial for the safety of both mothers and babies.^14,18^ BP values during pregnancy have been shown to be associated with a continuous inverse effect on foetal growth.^19^ Failing to assess and diagnose HDPs may result in premature birth, still birth, intrauterine death, intrauterine growth restriction and termination of pregnancy.^11,22,23^

The majority of pregnancy-related care is delivered by midwives at MOUs based at the primary healthcare level.^6^ It is thus critical that midwives have the required knowledge and skills to function independently without the support of a doctor and are able to refer to the next level of care when required. An early diagnosis could improve the pregnancy outcome because improved maternal and foetal monitoring would lead to earlier detection of clinical signs of the disease and, where necessary, medication can be given.^24^

### Strengths and limitations

This study was limited to MOUs at the PHC level and restricted to a particular district in the Eastern Cape. The second and third levels of healthcare services and private hospitals were excluded from the study.

The strengths of this study were that the return rate was 100% and only registered midwives practising in MOUs at the PHC level were included in the study. Furthermore, the study was quantitative, and the midwives were tested with a multiple-choice questionnaire.

### Implications or recommendations

The implementation of the curriculum of the new bachelor’s degree in general nursing and midwifery, in which the midwifery course is 1 year in duration, should be expedited to support a quality and safe midwifery practice.^26^

In addition, midwives in charge of MOUs should be equipped with a post-graduate qualification in advanced midwifery and neonatology so that they will be able to manage unforeseen obstetrical complications and the high-risk newborn. Despite the fact that continuous professional development in terms of the new *Nursing Act* 33 of 2005^7^ has not been promulgated as yet, providing evidence of attendance at workshops, conferences and refresher courses in obstetrics and midwifery should be compulsory. Midwives should not only be updated in theoretical knowledge, but their clinical skills should also be updated to ensure competence. Attendance at national programmes such as the Essential Steps in the Management of Obstetric Emergencies and the Basic Antenatal Care for PHC facilities should be made compulsory for all midwives.

The maternal and infant mortality rates will continue to increase if urgent measures are not implemented to ensure that midwives in charge of MOUs are qualified in advanced midwifery and neonatology, including continuous refresher courses, to ensure competence both in theory and in practice for all practicing midwives.

### Further research

To address the high infant and maternal mortality rates, further research is required on a national level on midwifery practice to enable appropriate interventions to be introduced nationally. In addition, research is recommended on other conditions in pregnancy such as diabetes, managing emergencies in obstetrics, obstetric shock, physiology, management and care during the second and third stages of labour and so on.

## Conclusion

In South Africa, the majority of pregnancy-related care is delivered by midwives at MOUs based at the primary healthcare level.^6^ Aggressive measures should thus be introduced and expedited action is required to improve the knowledge of midwives at the PHC level. HDPs are a major contributor to maternal deaths. Poor knowledge about these disorders and not being able to diagnose and assess a patient with HDPs accurately should not be tolerated for a quality and safe midwifery practice.
